# Perception and Practices Regarding Muscle Loss Protection in Childhood Cancer Patients: A National Survey of Chinese Pediatric Oncologists in China

**DOI:** 10.1155/jnme/8934452

**Published:** 2026-04-29

**Authors:** Feng Tian, Yali Han, Pengli Wang, Chencheng Xu, Qianjun Wan, Kejia Yang, Dapeng Jiang

**Affiliations:** ^1^ Department of Pediatric Surgery, Suzhou Wujiang District Children’s Hospital, Suzhou, China; ^2^ Department of Oncology (Ward 2), Shanghai Children’s Medical Center, Shanghai Jiao Tong University School of Medicine, Shanghai, China, shsmu.edu.cn; ^3^ Department of Pediatrics, Qingdao Municipal Hospital, Affiliated to University of Health and Rehabilitation Sciences, Qingdao, China, qdslyy.cn; ^4^ Department of Emergency, Maternal and Child Health Hospital of Hubei Province, Wuhan, China

**Keywords:** guideline, muscle loss protection, pediatric cancer, radiotherapy, sarcopenia

## Abstract

**Purpose:**

To investigate perception and practices regarding muscle loss protection (MLP) among Chinese pediatric oncologists in a nationwide survey.

**Methods:**

A nationwide online survey was conducted among pediatric oncologists using a self‐designed structured questionnaire assessing predominant roles, barriers, practices, attitudes, and knowledge. Behavior and attitude were measured using a 5‐point Likert scale. Barrier scores were based on classification: denial scores 3, uncertainty 2, and confirmation 1. Knowledge scores were calculated based on the number of correct responses (one point per correct answer). Binary logistic regression, ordinal logistic regression, and multiple linear regression models were applied to identify factors associated with knowledge, attitudes, behaviors, and composite scores related to MLP, and spatial analysis (Moran’s I) was used to evaluate geographic clustering.

**Results:**

Among 253 respondents, physicians, surgeons, physiatrists, and nutritionists were most trusted to lead MLP. Physicians, particularly hematologists, received the most support as the predominant specialists. While the absence of relevant guidelines and consensus was a significant barrier, the primary barrier was illness severity. Although the general attitude was positive, behavior and knowledge were far from satisfactory. Doctors in centers with ML assessment resources had significantly higher practice scores. Spatial analysis indicated regional variation in scores, with localized patterns of relatively higher and lower performance across provinces.

**Conclusion:**

Chinese pediatric oncologists demonstrate positive attitudes but concerning deficiencies in MLP practices and knowledge, with notable disparities primarily associated with institutional resources and regional context. The development of evidence‐based national guidelines is strongly recommended to standardize care and address these gaps.

## 1. Introduction

From the 1980s to the 2010s, the incidence of malignant tumors in children aged 0–14 increased from 124.0 to 140.6 per million children per year [1]. With advances in medical science, the 5‐year survival rate for childhood cancers has exceeded 80%. However, relying solely on the 5‐year survival rate to assess outcomes for children with cancer, particularly during their critical growth and development phase, is no longer adequate. We must adopt a more holistic and long‐term perspective in assessing childhood cancers. Furthermore, intensified treatments have increased the prevalence of long‐term side effects. Compared to the general population, survivors of childhood cancer continue to face an elevated risk of late mortality [2–4], including conditions such as cardiovascular diseases, hypertension, diabetes, underweight or obesity, bone mineral deficiency, and endocrine metabolic disorders, all of which are related to muscle health and function. Patients with muscle loss (ML) and fatigue experience weakened conditions that affect the frequency of hospitalizations, fat storage, immune function, growth and development, and psychological health [5, 6]. Recent studies have also shown that muscle deficits are a common phenomenon among survivors of childhood cancer [7], a concern that is garnering increasing attention.

In 2016, the International Classification of Diseases, 10th Revision, Clinical Modification (ICD‐10‐CM) recognized sarcopenia as a distinct disease, despite lacking a universally accepted, clear definition [8]. In 2018, the European Working Group on Sarcopenia in Older People revised the original definition, stating that “sarcopenia is a muscle disease (muscle failure) rooted in adverse muscle changes that accrue across a lifetime; sarcopenia is common among adults of older age but can also occur earlier in life” [9]. Research on sarcopenia has been primarily concentrated on elderly individuals or older adults with cancer and has reached some consensus conclusions, as well as identified novel therapeutic approaches that may be translated into clinical practice [10–12]. Despite updated concepts indicating that sarcopenia is not limited to old age, pediatric oncologists are increasingly aware of ML in pediatric cancer patients, yet there remains a dearth of guidelines and consensus tailored to pediatric sarcopenia.

This change and the current situation highlighted the need to explore sarcopenia in younger populations, particularly in the context of pediatric cancer. While research on pediatric cancer–related ML is scarce and narrow in scope, particularly with a significant absence of large‐scale randomized controlled trials, the concept of sarcopenia in pediatrics and its diagnosis did not reach a unified standard, and there is also a lack of uniformity in the criteria used for research [13], which could be concerned with pediatricians underemphasizing muscle loss protection (MLP). There is a lack of studies on ML of childhood cancer in China, and the clinical practices and perception of Chinese pediatricians regarding MLP remain unclear. Consequently, given China’s vast territory and the persistent imbalance in regional development, we embarked on this investigation to assess the current perception and practices of pediatricians across China concerning MLP in pediatric cancer patients, aiming to clarify the clinical practices of Chinese pediatricians in managing MLP for pediatric cancer patients, identify any potential issues, and explore the geographical variations in these practices across different regions.

## 2. Methods

### 2.1. Study Design

This study developed a pediatric oncologist‐specific questionnaire, drawing on surveys that assess healthcare professionals′ understanding of sarcopenia in aging patients [14, 15]. The purely textual electronic questionnaires created on the “Wenjuanxing” platform <https://www.wjx.cn> were anonymously distributed to members of the pediatric oncology‐related professions within the Chinese Anti‐Cancer Association via the WeChat platform. The completion of the questionnaire was estimated to take approximately 10 min, with all questions set as mandatory, ensuring that the form could only be submitted after all items had been addressed, and each participant was limited to one submission. Ethical approval for this study was obtained from the institutional review board (NO. 2024014).

The questionnaire comprised 16 items to capture demographic information and included 8 questions each to evaluate behaviors, attitudes, knowledge, barriers, and reasonable or predominant specialties regarding MLP in the clinical setting (Supporting 1). “Behavior” and “attitude” were assessed using a 5‐point Likert scale, with scores ranging from 1 to 5 corresponding to “never, seldom, sometimes, usually, always” and “completely disagree, disagree, uncertain, agree, completely agree,” respectively. The sum of the scores for the eight questions was designated as the “behavior score” and “attitude score.” Knowledge was assessed using eight true/false questions covering key domains of MLP, including causes, assessment, and management of ML in childhood cancer. One point was awarded for each correct response, whereas incorrect or uncertain responses were assigned zero points. The total Knowledge score was calculated by summing the scores across all eight items, with higher scores indicating greater MLP‐related knowledge. Barrier items were designed as reverse‐coded indicators of MLP implementation. Responses confirming the presence of a barrier were assigned 1 point, responses indicating uncertainty were assigned 2 points, and responses denying the presence of a barrier were assigned 3 points. The total Barrier score was calculated by summing the scores across all items, with higher scores indicating fewer perceived barriers and better overall performance. An additional question was included to identify the “primary barrier,” which respondents considered the most significant. The “predominant specialty” section mirrored the “barrier” section, presenting eight distinct disciplinary options and an additional question to pinpoint the predominant specialty, although without an associated scoring attribute. The total score was derived from the sum of scores in behavior, attitude, knowledge, and barriers, with higher scores in each category reflecting a more proactive approach from the respondents. Eligible participants for the survey were pediatric physicians and surgeons with a focus on pediatric oncology from all provinces of China, practicing at various levels of hospitals capable of providing pediatric tumor treatment.

Prior to nationwide dissemination, the questionnaire underwent content review by a multidisciplinary expert panel, including pediatric oncologists, surgeons, radiotherapists, and rehabilitation medicine specialists, to assess the relevance and clarity of each item within the context of MLP in childhood cancer. In addition, a pilot test was conducted among a small group of pediatric oncology–related physicians, and minor wording adjustments were made based on their feedback to improve interpretability and feasibility. Owing to the inclusion of several nonscale items, the questionnaire was not subjected to full psychometric validation.

### 2.2. Statistical Analysis

Data were analyzed using SPSS (Version 26.0) and GraphPad Prism (Version 8.0.2), with all *p* values calculated as two‐tailed and a significance level of *p* < 0.05. Demographic data served as the independent variable in regression analysis, while questionnaire responses, apart from the demographic details, served as both the dependent and secondary independent variables for distinct analyses. Since the raw questionnaire data were all categorical data except geographical information, chi‐square tests were initially employed to identify variables with potential statistical differences. Variables identified as potentially statistically different, along with various scores calculated, were subsequently included in univariate regression analysis. Finally, variables identified as statistically significant in the univariate analysis were included in the multivariate regression analysis. “Knowledge” was assessed using binary logistic regression analysis, and “behavior,” “attitude,” “barriers,” and “reasonable or predominant” were evaluated with ordinal logistic regression analysis. For models failing the parallel lines test and unordered models, multiple unordered logistic regression analyses were conducted, calculating the odds ratios (OR) and 95% confidence intervals (CI). All score variables were analyzed with multiple linear regression, confirming no significant multicollinearity (variance inflation factor < 5), and yielding the linear regression coefficients β along with their 95% CI. Spearman correlation analysis was performed to examine the relationships among Attitude, Behavior, Knowledge, and Barrier scores.

To facilitate the analysis of regression factors, respondents’ geographical information was categorized into four regions based on factors such as geographic location, administrative divisions, and economic levels: the Eastern region, including Beijing, Tianjin, Hebei Province, Shandong Province, Shanghai, Jiangsu Province, Zhejiang Province, Fujian Province, Guangdong Province, Hainan Province, Hong Kong Special Administrative Region, Macao Special Administrative Region, and Taiwan Province; the Northeast region, including Liaoning Province, Jilin Province, and Heilongjiang Province; the Central region, including Anhui Province, Shanxi Province, Henan Province, Hubei Province, Hunan Province, and Jiangxi Province; and the Western region, including Shaanxi Province, Chongqing Municipality, Sichuan Province, Gansu Province, Guizhou Province, Yunnan Province, Qinghai Province, Inner Mongolia Autonomous Region, Xinjiang Uygur Autonomous Region, Ningxia Hui Autonomous Region, Xizang Autonomous Region, and Guangxi Zhuang Autonomous Region. The spatial disparity of dependent variables was evaluated using Moran’s index.

## 3. Results

### 3.1. Demographic Information

Pediatricians from more than 60 cities across 27 provinces in China responded to the questionnaire, with 253 valid responses, constituting 72.3% of the total distributed. Among the participants, 45.8% were male and 54.2% were female. The specialties included 28.9% physicians, 17.4% hematologists, 47% surgeons, and 6.7% oncology surgeons. 49.3% had over 10 years of practice, and 64% held a master’s degree or above. More than half of the respondents were from the economically developed Eastern region, and the vast majority were nonreligious. Over 80% of the participants were from Grade III Level A teaching hospitals, which represent the highest hospital rating in China and indicate the most advanced medical resources. Subsequent levels include 3B, 2A, 2B, 1A, and 1B. However, the majority indicated that their departments had no more than five pediatric oncology specialists, 10 pediatric oncology beds, and admitted no more than 100 pediatric cancer patients annually. Details are shown in Table 1.

**TABLE 1:** Demographic information.

Subjects		*N*	%
Total		253	100

Sex	Male	116	45.8
	Female	137	54.2

Age group (year)	20–29	56	22.1
	30–39	93	36.8
	40–49	61	24.1
	≥ 50	43	17

Education background	Below bachelor’s degree	9	3.6
	Bachelor’s degree	82	32.4
	Master’s degree	106	41.9
	Doctor’s degree	56	22.1

Geographic position of hospital	The Eastern region	176	69.6
	The Northeast region	29	11.5
	The Central region	22	8.7
	The Western region	26	10.3

Level of hospital	3A	204	80.6
	3B	40	15.8
	2A and below	9	3.6

Teaching hospital	Yes	203	80.2
	No	50	19.8

Specialization	Physician	73	28.9
	Hematologist	44	17.4
	Surgeon	119	47
	Oncology surgeon	17	6.7

Year of experience	< 5 years	71	28.1
	5–10 years	57	22.5
	11–20 years	57	22.5
	> 20 years	68	26.9

Religious belief	Yes	14	5.5
	No	239	94.5

Number of colleagues specialized in pediatric cancer in the same department	< 5	143	56.5
	5–10	46	18.2
	11–20	16	6.3
	> 20	48	19

Number of beds for cancer patients	≤ 10	154	60.9
	11–20	32	12.6
	21–50	35	13.8
	51–80	19	7.5
	≥ 80	13	5.1

Number of cancer patients received per year	< 100	176	69.6
	100–300	41	16.2
	300–500	16	6.3
	> 500	20	7.9

Radiotherapy available in hospital	Yes	74	29.2
	No	179	70.8

Bone marrow transplantation available in hospital	Yes	138	54.5
	No	115	45.5

Pediatric cancer patients in relatives	Yes	5	2
	No	248	98

Muscle loss medicine department or specialist in hospital	Yes	51	20.2
	No	202	79.8

### 3.2. Who Should Be Responsible for or Predominant MLP

Among the eight surveyed specialties: physician and hematologist, surgeon and oncology surgeon, radiotherapist, nutritionist, physiatrist, endocrinologist, orthopedist, and traditional Chinese medical doctor (TCMD), there were a total of 1377 affirmative responses, with an average of 5.443 “yes” selections per respondent. Averages were 4.554 for doctoral degree holders, 5.377 for those with a master’s degree, and 6.159 for bachelor’s degree respondents. Physicians, particularly hematologists, received the most support as both the reasonable (85.4%) and the predominant (40.7%) specialists. A total of 88.9% of physicians and hematologists believed they should take the primary role, compared to 78.7% of surgeons and oncology surgeons. However, only 51.3% of physicians and hematologists and 28.7% of surgeons and oncology surgeons saw themselves as the predominant specialists. Physiatrists were supported by 85.0% of respondents, and nutritionists garnered 83.0% support in the “reasonable” category, both exceeding the surgeons’ 77.1%. In the “predominant” category, however, physiatrists received 18.2% of the votes and nutritionists received 13.4%, both lower than surgeons’ 20.6%. Table 2 shows the votes for this section. Factors primarily influencing “Who should be responsible for MLP” included educational level, barriers, and age groups. The primary determinants influencing “Who should predominate MLP” encompassed specialty, gender, behavior, and the hospital’s capacity to perform bone marrow transplantation. Compared with surgeons, physicians were significantly more likely to endorse radiotherapists as the predominant leaders of MLP (OR = 1.994 × 10^4^, 95% CI: 363–1.096 × 10^6^, *p* < 0.001). Full effect sizes are shown in Figures 1(a) and 1(b).

**TABLE 2:** Votes for reasonable and predominant.

	Reasonable				Predominant	
	Yes	%	No	Uncertain	*N*	%
Physician and hematologist	216	85.4	15	22	103	40.7
Surgeon and oncology surgeon	195	77.1	26	32	52	20.6
Radiotherapist	135	53.4	72	46	6	2.4
Nutritionist	210	83	18	25	34	13.4
Physiatrist	215	85	17	21	46	18.2
Endocrinologist	114	45.1	77	62	0	0
Orthopedist	183	72.3	39	31	11	4.3
TCMD	109	43.1	72	72	1	0.4

Abbreviation: TCMD, traditional Chinese medical doctor.

FIGURE 1Forest plot: (a) relative factors of “who should be reasonable for MLP” (OR and 95% CI); (b) relative factors of “who should predominant MLP” (OR and 95% CI). MLP: muscle loss protection. TCMD: traditional Chinese medical doctor.

**Figure:**
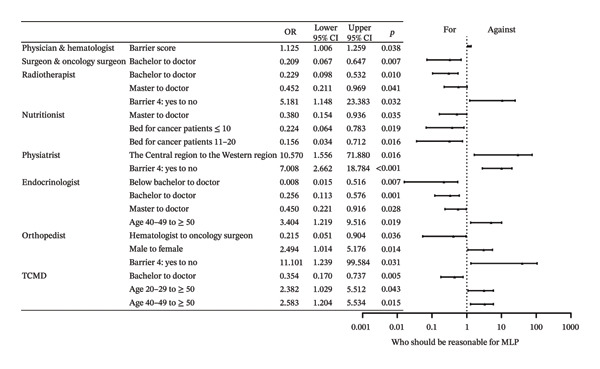
(a)

**Figure:**
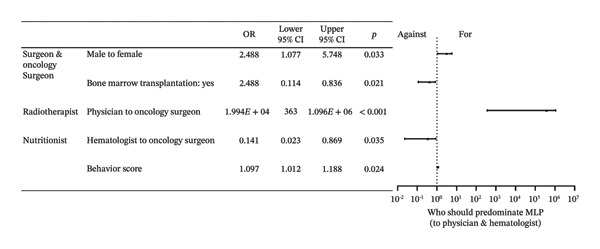
(b)

### 3.3. Barrier and Primary Barrier Evaluation

Barriers 1–8 were as follows: (1) Treatment cannot be delayed. (2) Families are unable to afford. (3) Parents don’t consider it important. (4) Doctors don’t consider it important. (5) There is no one or no place to carry out the relevant work. (6) There are no relevant guidelines or consensus. (7) Clinical workload hinders communication. (8) Informing parents may lead to refusal of further treatment. A total of 917 responses confirming the presence of barriers were recorded, with an average of 3.625 per individual. The averages were 3.589 for physicians, 3.932 for hematologists, 3.571 for surgeons, and 3.353 for oncology surgeons. Among all items, Barrier 1 was the most prevalent, accounting for 71.1%, followed by Barrier 6 at 62.8%. The primary barrier was Barrier 1, representing 25.7%. Table 3 presents the voting results for this section. The primary factors influencing the barriers included the capabilities and configurations of the hospital, as well as the selection of the predominant MLP specialty. The primary determinants of “primary barrier” were found to be educational attainment, specialty, geographical region, and partial scores. Full effect sizes are shown in Figures 2(a) and 2(b).

**TABLE 3:** Votes for barrier and primary barrier.

	Barrier				Primary barrier	
	Yes	%	No	Uncertain	*N*	%
Barrier 1	180	71.1	13	60	65	25.7
Barrier 2	128	50.6	50	75	35	13.8
Barrier 3	99	39.1	84	70	17	6.7
Barrier 4	41	16.2	160	52	22	8.7
Barrier 5	149	58.9	59	45	46	18.2
Barrier 6	159	62.8	47	47	52	20.6
Barrier 7	91	36	112	50	5	2
Barrier 8	70	27.7	102	81	11	4.3

FIGURE 2Forest plot: (a) relative factors of “barrier” (OR and 95% CI); (b) relative factors of “what is the primary barrier for MLP” (OR and 95% CI). Barrier 4: doctors don’t think it’s important. Groups marked with “^∗^” was not suitable for multiple ordinal logistic regression, and multiple logistic regression was used instead, and the choice in the middle, which meant there was no obvious preference, was used as a reference to assess the preferences of the respondents, so “for or against” could not be the reference for it. ML: muscle loss. MLP: muscle loss protection.

**Figure:**
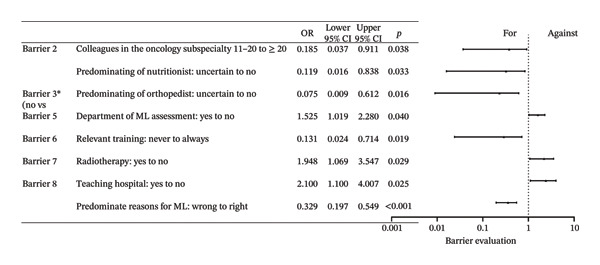
(a)

**Figure:**
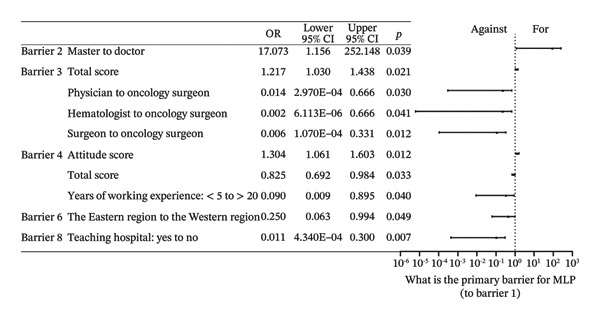
(b)

### 3.4. Behavior Evaluation

Behaviors 1–8 encompassed clinical practices regarding MLP in children: (1) Initiating discussions with patients or their families about MLP; (2) providing patients or their families with MLP‐related materials; (3) assessing ML during clinical treatment; (4) willing to conduct research on pediatric ML; (5) referring patients to rehabilitation, nutrition departments, or specific units; (6) consulting with specialists about potential ML issues including nutritionists and physiatrists; (7) participating in professional training related to pediatric MLP, and (8) showing interest in guidelines or advancements related to pediatric MLP. Over 80% of respondents typically performed poorly in Behavior 2, and over 70% in Behavior 1. Fewer than 30% demonstrated satisfactory performance in Behavior 3. Although over 50% of respondents indicated they would perform well in Behavior 5, 55.7% indicated that they hardly did well in Behavior 6. Furthermore, only about 20% were aware of the departments assessing ML in their own hospitals. About 30% regularly participated in MLP‐related training, and only 20.1% showed good performance in Behavior 8. A multitude of factors were associated with behavior, with the primary influences being the presence of the department of ML assessment in hospitals, years of working experience, educational attainment, and the busyness of clinical work. Full effect sizes are shown in Figure 3(a).

FIGURE 3Forest plot: (a) relative factors of “behavior” (OR and 95% CI); (b) relative factors of “attitude” (OR and 95% CI); (c) relative factors of “knowledge” (OR and 95%CI); (d) relative factors of all types of “scores” (β and 95% CI). Groups marked with “^∗^” was not suitable for multiple ordinal logistic regression, and multiple logistic regression was used instead, and the choice in the middle, which meant there was no obvious preference, was used as a reference to assess the preferences of the respondents, so “for or against” could not be the reference for it. Barrier 1: Treatment cannot be delayed. Barrier 4: Doctors don’t think it’s important. Barrier 6: There are no relevant guidelines or consensus. Barrier 7: Clinical workload hinders communication. ML: muscle loss. MLP: muscle loss protection.

**Figure:**
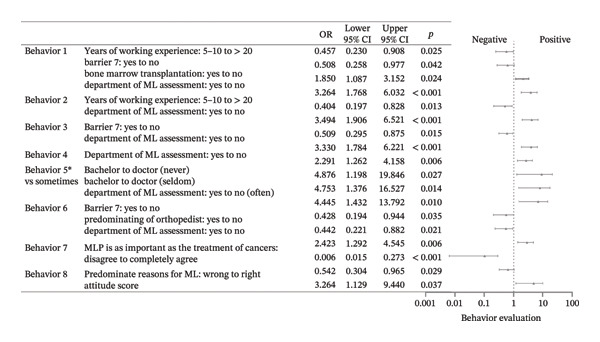
(a)

**Figure:**
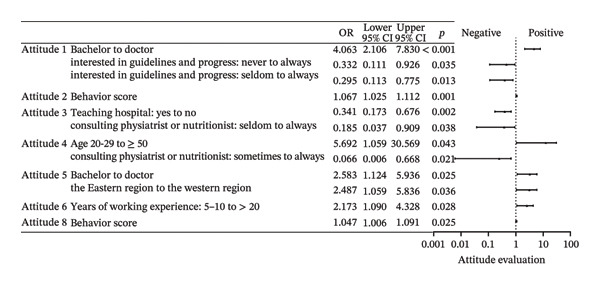
(b)

**Figure:**
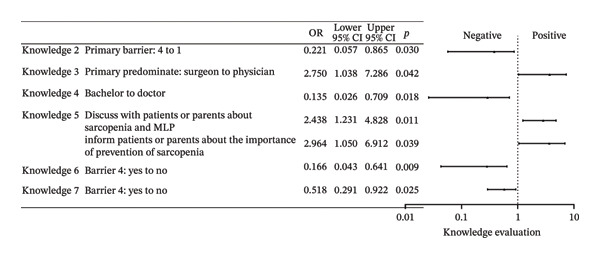
(c)

**Figure:**
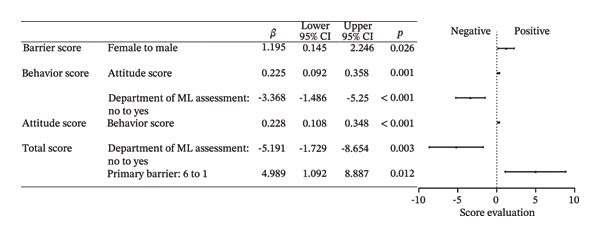
(d)

### 3.5. Attitude Evaluation

Attitudes 1–8 mainly concern: (1) Emphasizing the significance of ML as a risk in cancer treatment, (2) discussing the impact of tumor treatment with patients and parents, (3) emphasizing early prevention of ML, (4) equating the importance of MLP with primary tumor treatment, (5) concerning ML in patients even with a poor prognosis, (6) the severity of ML in higher tumor stages, (7) the necessity for pediatric oncology staff training on MLP, and (8) the necessity for guidelines and consensus on pediatric MLP in China. Respondents generally demonstrated a positive attitude. Specifically, 70.8% considered ML a risk requiring special emphasis in cancer treatment, 82.2% agreed on the necessity of informing and emphasizing to parents, and 86.6% affirmed the importance of early prevention. A total of 76.3% believed that MLP is as important as treating the primary tumor, and 79.8% thought that MLP should be addressed even in patients with poor prognosis. Additionally, 79.8% agreed that higher tumor stages have a more severe impact on ML. The proportion of respondents who believe in the need for training on the prevention and treatment of pediatric ML reached 87.4%, and the highest percentage, 88.9%, expressed a positive attitude toward the development of guidelines or consensus for pediatric MLP in China. Factors associated with attitudes primarily encompassed educational qualifications, geographical region, and certain behaviors. Full effect sizes are shown in Figure 3(b).

### 3.6. Knowledge Evaluation

The content of knowledge mainly pertained to: the existence of guidelines or consensus (Knowledge 1), the causes of ML (Knowledge 2–3), the relationship between tumor‐related ML and fat (Knowledge 4), ML and tumor complications (Knowledge 5), the assessment level of muscle and fat (Knowledge 6), treatment (Knowledge 7), and evaluation (Knowledge 8). Unfortunately, the overall correct rate for any knowledge question did not reach 50%. 42.7% of respondents understood that ML is not due to surgical resection, and 33.6% recognized that pediatric ML is related to prognosis and complications of cancer treatment. Only about 15% were relatively clear about the main causes of ML, and nearly 90% of people were unaware that there were no guidelines for MLP in pediatric cancer patients. Over 90% of respondents were unfamiliar with more complex knowledge regarding fat changes during ML, assessment levels by imaging examination, drug treatment, and reference thresholds for the assessment of pediatric ML. Respondents from 3A hospitals had slightly higher correct rates in some questions, but no significant statistical differences were found in the multivariate regression analysis. Factors associated with knowledge, apart from educational background, were largely subjective, with primary factors being behavior and assessments of “barrier 4.” Individuals who recognized the existence of Barrier 4 showed a tendency toward poorer knowledge performance. Full effect sizes are shown in Figure 3(c).

### 3.7. Scores

The average and median scores for each rating item are presented in Table 4. The scores for behavior and knowledge were relatively low, as both the average and median scores did not reach half of the theoretical maximum. In contrast, the average and median scores for attitudes exceeded 80% of the upper limit. As the Barrier score was reverse‐coded, higher values indicated fewer perceived barriers and better overall performance. Factors that primarily influenced the scores included the department of ML assessment and barriers without guidelines or consensus. Details are shown in Figure 3(d). Spearman correlation analysis revealed weak but statistically significant positive associations between Attitude and Behavior scores and between Knowledge and Behavior scores, whereas no significant correlation was observed between Attitude and Knowledge. Barrier scores were not significantly correlated with other domains (Table 5). Spatial autocorrelation analysis showed a dispersed global pattern for both total and behavior scores. Nevertheless, localized differences were observed across provinces, with relatively higher scores identified in Sichuan Province compared with some neighboring regions, demonstrating a certain degree of localized heterogeneity. Details are shown in Figure 4.

**TABLE 4:** The individual subproject scores and the final score.

	Barrier score	Behavior score	Attitude score	Knowledge score	Final score
Average score	14.85	17.76	32.6	1.28	66.49
Median score	15	17	32	1	66
Full score on theory	24	40	40	8	112

**TABLE 5:** Spearman correlation coefficients among attitude, behavior, knowledge, and barrier scores.

Variable	Behavior	Barrier	Attitude	Knowledge
Behavior	—	0.093	0.179^∗^	0.189^∗^
Barrier	0.093	—	−0.032	0.079
Attitude	0.179^∗^	−0.032	—	0.072
Knowledge	0.189^∗^	0.079	0.072	—

*Note:* Values represent Spearman’s rank correlation coefficients (*ρ*). Attitude, Behavior, Knowledge, and Barrier scores represent composite scores derived from the corresponding questionnaire domains.

^∗^ *p* < 0.05 (two‐tailed).

FIGURE 4Spatial autocorrelation analysis. (a) Aggregation pattern of the total score; (b) aggregation pattern of the behavior score.

**Figure:**
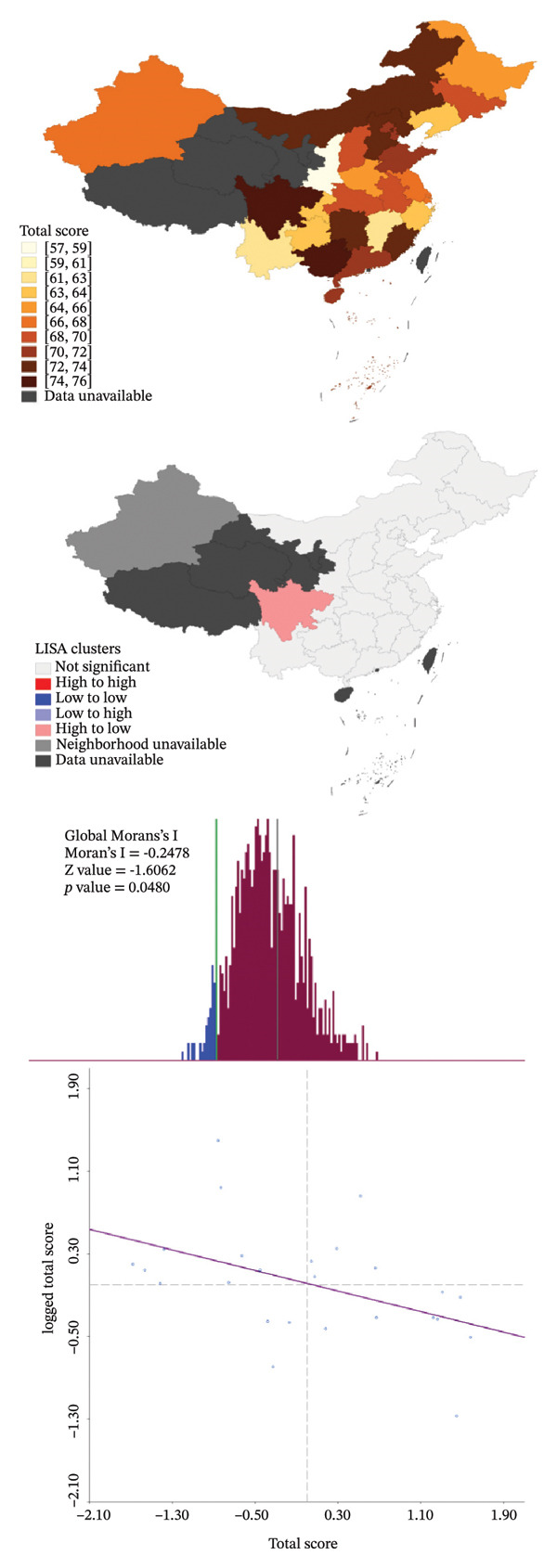
(a)

**Figure:**
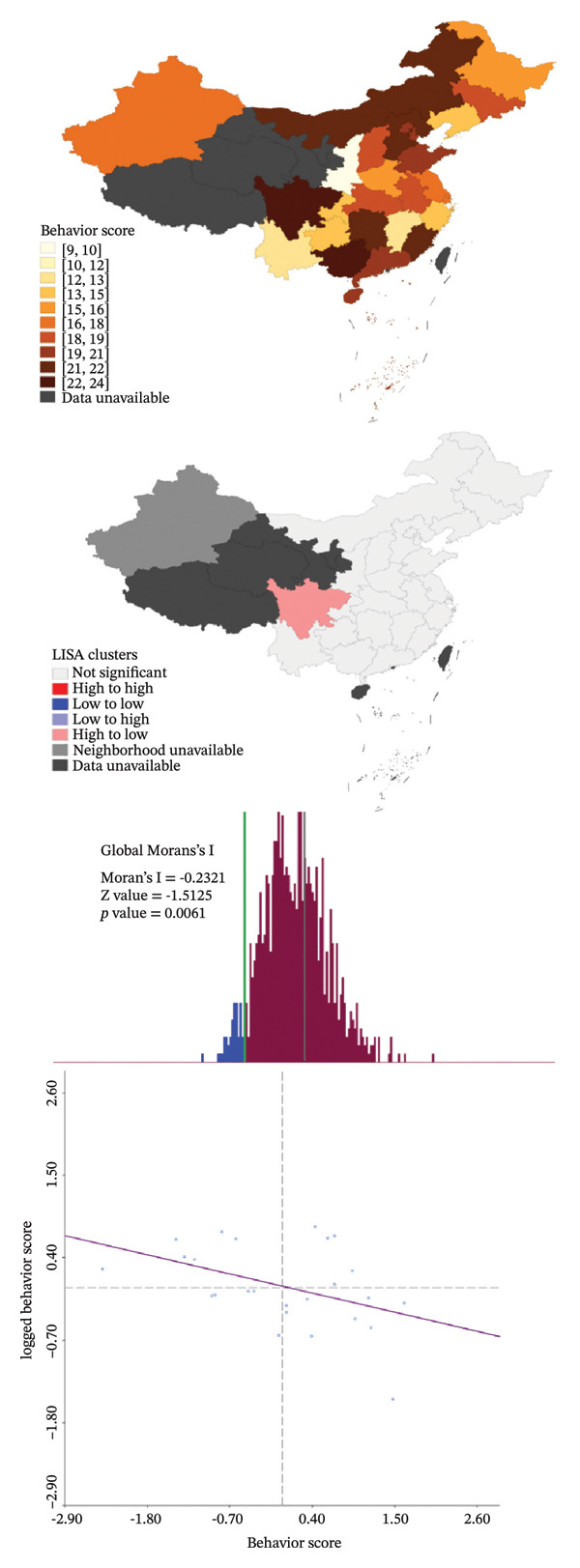
(b)

## 4. Discussion

The 5‐year and long‐term survival rates of pediatric cancer patients in China have gradually increased, approaching the levels of developed countries. To further enhance treatment outcomes, long‐term survival, and the quality of life in pediatric cancer patients, MLP merits attention [16–19]. Developed countries in Europe and America have conducted significant research on ML in survivors of childhood cancer, detailing long‐term muscle changes and various risk factors, including gender, chemotherapy, radiotherapy, and nutrition [20, 21]. To some extent, exercise and rehabilitation therapies have been proven effective [22]. Conversely, few Asian countries have a limited number of studies, often with small sample sizes, which may reflect a general lack of emphasis on this issue in Asia [23, 24]. Compared to the well‐established research in sarcopenia among the elderly, the field of ML associated with pediatric cancer and its protective measures remains largely unexplored. The academic community lacks not only unified guidelines and consensus on MLP in children with cancer but also a standardized approach for diagnosis and evaluation; even though there remains ongoing debate regarding several fundamental conceptual issues of pediatric ML and sarcopenia [13, 25–27]. In addition, researchers on pediatric ML must take children’s growth and development, neurocognitive status, and ethical issues into consideration, adding to the complexity of research [28]. This study reveals some current realities regarding pediatric MLP among Chinese pediatricians in clinical practice, including a wide range of disciplines involved, various clinical barriers, a generally positive attitude, yet a certain degree of deficiency in behavior and knowledge, which may also be related to geographical regions.

Physicians and hematologists leading chemotherapy were considered the predominant specialties in MLP, and respondents from centers capable of performing bone marrow transplantation leaned toward the belief that surgeons should not hold this position. A certain degree of variation among respondents from different specialties in assessing the predominant role in MLP potentially affecta how they identify and manage patients′ MLP needs. Interestingly, although radiotherapists received fewer overall votes, compared to surgical respondents, physicians strongly advocated for radiotherapists to be the predominant MLP leaders rather than hematologists, indicating a recognition of the significant impact of radiotherapy on ML. This observation not only reflected a perceived lack of self‐confidence in their own leadership capabilities but also highlights the underappreciated impact of radiotherapy on ML, potentially as significant as chemotherapy, according to physicians who lead chemotherapy regimens. Radiotherapy, a primary treatment for over 50% of cancer patients, can have varying side effects influenced by radiotherapy techniques, patient age, and treatment sites [29, 30]. The impact of radiotherapy on the tumor microenvironment is complex and multifaceted, involving various levels such as tumor vasculature, cells of the immune system, and hypoxic responses, which may exert counterproductive effects on tumor treatment [31]. In addition to affecting the structure and function of local tissues and organs, radiotherapy can also impact children’s growth and development, endocrine function, skeletal muscles, and the risk of secondary tumors, and side effects such as muscle atrophy and weakness may persist for many years [32]. Compared to adults, children may exhibit different changes in muscle fiber types following radiotherapy, and these alterations could potentially impact the functionality and metabolic characteristics of the muscles [33]. A German study noted a higher incidence of side effects in patients treated with radiotherapy in neighboring countries, suggesting that nontraditional factors may influence radiotherapy side effects, with mechanisms yet to be fully understood [34]. These findings highlight the need for increased focus and research on the impact of radiotherapy on pediatric cancer patients by radiotherapists and oncologists [35–38]. Emerging radiotherapy methods like FLASH therapy hold the potential to further reduce side effects, yet their clinical application and safety in pediatric patients necessitate further research [39, 40]. While the clinical impact of radiotherapy on ML is increasingly recognized at a conceptual level, its role in structured MLP leadership remains underdefined. This misalignment, together with limited resources, highlights the need to better delineate multidisciplinary responsibilities and to strengthen pediatric‐specific education, training, and collaboration pathways. Such efforts may help translate conceptual support for radiotherapy‐related and multidisciplinary contributions into consistent and effective MLP implementation in pediatric oncology practice.

Apart from Barrier 1, that the disease itself cannot delay treatment, the lack of relevant clinical guidelines or consensus was identified as the most significant barrier. Those who never participated in relevant training were more inclined to regard it as a barrier. Compared to respondents from the Eastern region, those from the Western region were more affected by the lack of guidelines or consensus. Due to the uneven development of healthcare in China, respondents from the Western region often received less professional guidance, which may have contributed to comparatively lower performance in behavior and knowledge. The finding that nearly 90% of respondents were unaware that no pediatric‐specific MLP guidelines currently exist likely reflects not a complete absence of guidance awareness, but rather a perceived existence of applicable recommendations. In clinical practice, adult oncology or sarcopenia‐related guidelines may be informally extrapolated to pediatric settings, leading clinicians to assume that formal guidance is already available. This highlights a gap between perceived and actual guideline availability and underscores the need for clearly articulated, pediatric‐specific MLP recommendations. The survey also indicated that respondents were most enthusiastic about the establishment of guidelines for the MLP against sarcopenia among Chinese children with cancer. Those who expressed a more urgent need for guidelines and consensus also had higher total scores. ML is prevalent among children and adolescents with cancer and is associated with various diseases, including acute lymphoblastic leukemia, liver and kidney diseases, gastrointestinal diseases, high‐risk hepatoblastoma, and neuroblastoma [6]. Although sarcopenia has become an important marker for assessing patient prognosis in adults, research on sarcopenia in children has not yet reached a clear consensus [41]. Sarcopenia in children lacks established diagnostic criteria and methods for assessing muscle mass and function, as well as reference values for different age groups. The existing research is relatively limited, and the relationship between inflammation and sarcopenia remains unclear [28]. These factors make the development of guidelines more challenging. Especially in the resource‐limited regions with relatively underdeveloped economic and medical infrastructure, respondents tended to choose TCMDs to be reasonable for MLP more frequently, reflecting a potential lack of MLP methods due to the absence of guidelines, although there is no statistically significant difference.

Consistent with the dispersed global Moran’s I results, local indicators of spatial association (LISA) revealed localized “High‐to‐Low” patterns confined to a limited number of provinces, rather than broad regional aggregation. These findings indicate that the observed spatial variation reflects province‐level heterogeneity rather than a coherent regional gradient. Although relatively higher total and behavior scores were observed in Sichuan Province, the LISA pattern suggests a high‐value outlier surrounded by lower performing neighboring regions, and there is currently no evidence to support the presence of specific regional initiatives or policy‐driven interventions related to MLP in pediatric oncology.

As a major economic and medical center in Western China, Sichuan Province hosts a concentration of pediatric oncology resources, which may partly explain the relatively higher scores observed. In contrast, several neighboring provinces in Western China, including Qinghai, Guizhou, Guangxi, and Yunnan, have comparatively limited pediatric medical infrastructure, which may contribute to lower levels of MLP‐related practice despite generally positive attitudes. This pattern is consistent with a potential “siphoning effect,” whereby specialized medical resources are concentrated in regional centers, while surrounding areas experience comparatively reduced clinical capacity. These findings highlight persistent regional disparities in pediatric oncology capacity and implementation across Western China, rather than the impact of identifiable regional programs. They further underscore the need to develop pediatric‐specific MLP guidelines and diagnostic or therapeutic protocols, alongside targeted training and capacity‐building strategies, to enhance the professional competencies of pediatric oncologists in resource‐limited regions.

Respondents from centers with dedicated ML assessment departments demonstrated superior performance in almost all the behavioral survey aspects, with consistent results in both behavior and total scores. Unfortunately, despite a significant portion of the surveyed population being from the Eastern region, only about 20% of respondents had access to hospital departments or specialists for ML assessment. ML or sarcopenia in children is not an aging‐related issue. The unique characteristics of children make it more difficult to carry out assessments or treatments requiring active cooperation. Sarcopenia can also impact the assessment of diseases such as cardiovascular diseases, liver and kidney diseases, endocrine functions, organ transplantation, and secondary tumors [42–44], and its own potential effects add to the complexity of these assessments. Assessing sarcopenia in children necessitates the development of standardized, nationally relevant survey methods and a multidisciplinary approach to comprehensively evaluate muscle strength, mass, physical performance, diet, and nutrition. Such an assessment likely involves collaboration across diverse medical fields, educational institutions, and health insurance sectors, though it is not restricted to these areas [45]. Currently, the pediatric community in China has not accorded sufficient emphasis on fields such as radiotherapy, nutrition, and physical therapy, with only a few top‐tier children’s hospitals achieving a comparatively comprehensive development of multidisciplinary teams and departmental construction. Moreover, pediatrician resources in China are extremely scarce, with a significant shortage affecting the healthcare system [46]. This study also indicated that the heavy workload in clinical practice had a certain negative effect on the behavior of pediatricians. These findings underscore the need for improved management of ML in pediatric cancer patients in China, highlighting the necessity for a multidisciplinary, comprehensive approach to MLP in children with cancer. Recent studies have indicated that newly developed auxiliary programs can be implemented in outpatient clinics across various countries to assess sarcopenia in the elderly [47]. Confronted with the intricacies of multidisciplinary development, as well as the unique evaluation methods required for children, employing auxiliary programs might be an alternative approach that could rapidly extend benefits broadly, especially in enhancing the medical standards in relatively underdeveloped regions.

76.3% of the respondents considered MLP as crucial as the treatment of the primary tumor, while only 19.8% disagreed that economic burden poses a barrier to MLP in pediatric cancer patients. Although there was no significant statistical association between them (*p* = 0.056), it is an undeniable fact that economic hardships can indeed amplify family stress. Globally, less than 40% of children with cancer received adequate diagnosis and treatment, with the majority residing in high‐income countries [4]. A 2022 national study in China on children and adolescents with cancer revealed that family financial burden can diminish the ability of children with cancer to access medical services, leading to delays and interruptions in treatment, affecting psychological and emotional well‐being, and restricting education and social interactions, thereby impacting the overall health of pediatric cancer patients. Additionally, seeking medical treatment across regions is highly prevalent in China, as patients often travel to more medically and economically developed areas for medical care, incurring additional expenses that further exacerbate the financial burden on parents and subsequently affect the treatment and recovery of children with cancer [48]. A study conducted in Jordan has also confirmed that economic issues can impose an additional burden on parents during the treatment process [49]. To alleviate the financial burden on parents and enhance the therapeutic outcomes and MLP engagement in pediatric cancer patients, the government needs not only more comprehensive health insurance policies but also to take corresponding measures to address the overconcentration of regional medical resources and to improve medical infrastructure in underdeveloped regions.

The feasibility of implementing comprehensive MLP strategies in pediatric oncology should be considered in the context of existing resource constraints. In many clinical settings, particularly in resource‐limited regions, the availability of specialized personnel and structured multidisciplinary teams may be restricted. Therefore, MLP implementation should be approached pragmatically, with an emphasis on stepwise integration into existing care pathways rather than the creation of parallel or resource‐intensive programs. Rather than requiring universal, high‐intensity multidisciplinary involvement, a risk‐stratified and needs‐based approach may allow key MLP components—such as early screening, basic nutritional assessment, and functional monitoring—to be incorporated into routine practice. Targeted multidisciplinary input can then be prioritized for high‐risk patients. Such an approach may help balance clinical benefit with economic and workforce considerations, thereby enhancing the real‐world applicability and sustainability of MLP strategies in pediatric oncology.

To our knowledge, this is the first national survey in China to investigate pediatric oncologists’ perceptions and practices regarding MLP in childhood cancer. Nevertheless, several limitations should be acknowledged. First, although the self‐designed questionnaire was reviewed by a multidisciplinary expert panel and pilot tested prior to formal distribution, it was not subjected to full psychometric validation owing to the inclusion of nonscale items. Consequently, the precision and reproducibility of the quantitative scores may be limited. Second, the relatively small sample size, together with a questionnaire retrieval rate of approximately 75%, may have introduced a certain degree of response bias. Moreover, the study sample was predominantly drawn from the Eastern region of China and from Grade III Level A teaching hospitals, reflecting the perceptions and practices of pediatric oncologists working in relatively resource‐rich centers. Therefore, our findings may overestimate the overall level of awareness and implementation of MLP in pediatric oncology across China, particularly in lower‐tier or rural hospitals, and caution is warranted when extrapolating these results at the national level. Finally, this study focused exclusively on self‐reported perceptions of pediatric oncologists, without incorporating objective assessments from other healthcare professionals or comprehensive evaluations from patients and families, which may limit the objectivity of findings related to cognition and clinical behavior. Future studies incorporating a broader range of healthcare providers, as well as patients and parents, are warranted to enhance the generalizability of these findings.

## 5. Conclusion

Despite generally positive attitudes toward MLP among Chinese pediatric oncologists, notable deficiencies persist in clinical practice, particularly with respect to behavior and knowledge. These gaps appear to be primarily associated with institutional capacity and resource availability. The findings suggest that limited awareness of pediatric cancer–related ML, insufficient clinical integration of MLP, and underdeveloped multidisciplinary collaboration frameworks remain key challenges in current practice. This survey underscores the need for strengthened pediatric‐specific research, clearer disciplinary development, and the establishment of evidence‐based national guidelines for MLP in children with cancer. Such efforts may facilitate more consistent implementation of MLP strategies and support the development of feasible, collaborative care models across diverse clinical settings in China.

## Funding

The authors declare that financial support was received for the research, authorship, and/or publication of this article. This work was supported by the Suzhou Young Health Talents “National Mentorship” Training Project (Qngg2024059), Wujiang District “Science & Education Promoting Health” Project (WWK202622), and Shanghai Hospital Development Center Foundation (SHDC12024128).

## Conflicts of Interest

The authors declare no conflicts of interest.

## Supporting Information

Supporting 1: English‐translated version of the survey questionnaire. (The original instrument was developed and administered in Mandarin Chinese.)

## Supporting information


**Supporting Information** Additional supporting information can be found online in the Supporting Information section.

## Data Availability

The data that support the findings of this study are available on request from the corresponding author. The data are not publicly available due to privacy or ethical restrictions.
