# Supplementary material for: Perception and Practices Regarding Muscle Loss Protection in Childhood Cancer Patients: A National Survey of Chinese Pediatric Oncologists in China
Source: J Nutr Metab. 2026 Apr 29;2026:8934452. doi: 10.1155/jnme/8934452 (PMC13126427; doi:10.1155/jnme/8934452)
Supplement: Supplementary file 1 — Supporting Information Additional supporting information can be found online in the Supporting Information section. [file JNME-2026-8934452-s001.docx]

**The final data results will be published in the form of research, and any personal information will be kept strictly confidential.**

1. Sex:

| ○male | ○female |
| --- | --- |

2. Age groups (years):

| ○20-29 | ○30-39 | ○40-49 | ○over 50 |
| --- | --- | --- | --- |

3. Education background:

| ○Below bachelor ○Bachelor ○Master ○Doctor |
| --- |

4.Which city your hospital belongs to:

_________________________________

5. Level of your hospital

| ○3A ○3B ○2A ○2B ○1A or 1B |
| --- |

6. Teaching hospital:

| ○yes ○no |
| --- |

7. Specialty:

| ○physician ○hematologist ○surgeon ○oncology surgeon |
| --- |

8. Working experience (years):

| ○＜5 ○5-10 ○11-20 ○＞20 |
| --- |

9. Religion belief:

| ○no ○Buddhist ○Christian ○Mohammedan ○others |
| --- |

10. How many medical staff in your department specialize in pediatric oncology:

| ○＜5 ○5-10 ○11-20 ○＞20 |
| --- |

11. How many pediatric oncology patient beds are there in your department:

| ○≤10 ○11-20 ○21-50 ○51-80 ○≥80 |
| --- |

12. How many pediatric oncology patients does your department treat annually:

| ○＜100 ○100-300 ○300-500 ○＞500 |
| --- |

13. Is your hospital capable of performing pediatric radiotherapy:

| ○yes ○no |
| --- |

14. Is your hospital capable of performing pediatric bone marrow transplantation:

| ○yes ○no |
| --- |

15. Have you or any of your close relatives ever had pediatric malignant tumors:

| ○yes ○no |
| --- |

16. Does your hospital have any departments or doctors that assess muscle loss:

| ○yes ○no |
| --- |

17. You will initiate discussions with the patient or family about the effects of muscle loss and interventions.

| ○never ○seldom ○sometimes ○usually ○always |
| --- |

18. You will provide the patient or family with information on how to conduct muscle loss preservation.

| ○never ○seldom ○sometimes ○usually ○always |
| --- |

19. You will assess the patient for during treatment and follow-up visits.

| ○never ○seldom ○sometimes ○usually ○always |
| --- |

20. Your team is willing to conduct research on muscle loss in children.

| ○never ○seldom ○sometimes ○usually ○always |
| --- |

21. You would refer a patient with issues related to muscle loss to physiatrist, nutritionist, or a specific unit.

| ○never ○seldom ○sometimes ○usually ○always |
| --- |

22. You will consult a physiatrist or nutritionist about potential muscle loss.

| ○never ○seldom ○sometimes ○usually ○always |
| --- |

23. You will participate in professional training related to muscle loss in children.

| ○never ○seldom ○sometimes ○usually ○always |  |
| --- | --- |

24. You are interested in guidelines or advances related to muscle loss in children.

| ○never ○seldom ○sometimes ○usually ○always |
| --- |

25. You identify barriers to the implementation of prevention and treatment of muscle loss in pediatric cancer patients.

|  | Yes | No | Uncertain |
| --- | --- | --- | --- |
| Treatment of tumor cannot be delayed | ○ | ○ | ○ |
| Parents can’t afford it | ○ | ○ | ○ |
| Parents don't think it's important | ○ | ○ | ○ |
| Doctors don't think it's important | ○ | ○ | ○ |
| There is no place/person to do the work | ○ | ○ | ○ |
| There are no relevant clinical practice guidelines or consensus | ○ | ○ | ○ |
| Busy clinical work affects communication with parents | ○ | ○ | ○ |
| Disclosure may lead parents to refuse subsequent treatment | ○ | ○ | ○ |

26. Which is the primary barrier:

| ○Treatment of tumor cannot be delayed |
| --- |
| ○Parents can’t afford it |
| ○Parents don't think it's important |
| ○Doctors don't think it's important |
| ○There is no place/person to do the work |
| ○There are no relevant clinical practice guidelines or consensus |
| ○Busy clinical work affects communication with parents |
| ○Disclosure may lead parents to refuse subsequent treatment |

27. The possibility of muscle loss due to treatment of malignancy is a risk that needs to be particularly emphasized during treatment.

| ○completely disagree ○disagree ○uncertain ○agree ○completely agree |
| --- |

28. Patients and parents need to be informed and discussed with them about the muscle and mobility damage caused by cancer treatment.

| ○completely disagree ○disagree ○uncertain ○agree ○completely agree |  |
| --- | --- |

29. Doctors treating malignant tumors need to inform patients and parents about the importance of early prevention of muscle loss.

| ○completely disagree ○disagree ○uncertain ○agree ○completely agree |
| --- |

30. Preventing and treating muscle loss is as important as treating the primary malignancy.

| ○completely disagree ○disagree ○uncertain ○agree ○completely agree |
| --- |

31. Doctors should pay attention to muscle loss even in patients with poor prognosis.

| ○completely disagree ○disagree ○uncertain ○agree ○completely agree |
| --- |

32. The effect of muscle loss is more severe in tumors with higher stages.

| ○completely disagree ○disagree ○uncertain ○agree ○completely agree |
| --- |

33. It is necessary for medical staff of pediatric oncology subspecialty to receive training in the prevention and treatment of muscle loss.

| ○completely disagree ○disagree ○uncertain ○agree ○completely agree |
| --- |

34. It is necessary for China to formulate some guidelines related to the prevention and treatment of muscle loss in children.

| ○completely disagree ○disagree ○uncertain ○agree ○completely agree |
| --- |

35. Who do you think should be reasonable for the preservation of muscle loss or muscle loss in children with malignant tumors.

|  | Yes | No | Uncertain |
| --- | --- | --- | --- |
| physician & hematology | ○ | ○ | ○ |
| surgeon & oncology surgeon | ○ | ○ | ○ |
| radiotherapist | ○ | ○ | ○ |
| nutritionist | ○ | ○ | ○ |
| physiatrist | ○ | ○ | ○ |
| endocrinologist | ○ | ○ | ○ |
| orthopedist | ○ | ○ | ○ |
| traditional Chinese medical doctor | ○ | ○ | ○ |

36. Who should predominate the preservation of muscle loss.

| ○physician & hematology |
| --- |
| ○surgeon & oncology surgeon |
| ○radiotherapist |
| ○nutritionist |
| ○ physiatrist |
| ○endocrinologist |
| ○orthopedist |
| ○traditional Chinese medical doctor |

37. There are clear guidelines or consensus for malignant tumor-related muscle loss in children at present.

| ○yes ○no |
| --- |

38. Excessive resection and destruction of muscle tissue during surgery is the main cause of muscle loss in children with tumors.

| ○yes ○no |
| --- |

39. The primary cause of muscle loss in children with malignant tumors is malnutrition.

| ○yes ○no |
| --- |

40. Children with muscle loss during the treatment of malignant tumors will have varying degrees of fat loss.

| ○yes ○no |
| --- |

41. Muscle loss in children is associated with prognosis, but not with complications of tumor treatment.

| ○yes ○no |
| --- |

42. The T12-L1 level is the best compromise site for assessing skeletal muscle, visceral adipose tissue, and subcutaneous adipose tissue, and has the highest correlation with whole body skeletal muscle and visceral fat.

| ○yes ○no |
| --- |

43. Vitamin D is one of the drugs proven to be effective in the treatment of muscle loss.

| ○yes ○no |
| --- |

44. There is no reference threshold for the evaluation of grip strength and 6-minute walk test in children aged 3-4 years at home and abroad.

| ○yes ○no |
| --- |
